# Occupational health among primary care workers: situational diagnosis

**DOI:** 10.47626/1679-4435-2022-751

**Published:** 2023-02-03

**Authors:** José Edmilson Silva Gomes, Shamyr Sulyvan Castro, Rodrigo Fragoso Andrade, José Jackson Coelho Sampaio, Patrícia Moreira Collares

**Affiliations:** 1 Gerência de Pós-Graduação em Saúde, Escola de Saúde Pública do Ceará, Fortaleza, CE, Brazil.; 2 Departamento de Fisioterapia, Universidade Federal do Ceará (UFC), Fortaleza, CE, Brazil.; 3 Programa de Pós-Graduação em Saúde Coletiva, UFC, Fortaleza, CE, Brazil.; 4 Departamento de Saúde Comunitária, UFC, Fortaleza, CE, Brazil.

**Keywords:** Worker’s health, primary health care, situational diagnosis, saúde do trabalhador, atenção primária à saúde, diagnóstico situacional

## Abstract

**Introduction:**

The field of workers’ health within the scope of the Brazilian Unified Health System needs to revitalize its space in terms of coordinating care in primary health care based on social determinants of health.

**Objectives:**

To contextualize and describe the health-related situational diagnosis of primary care workers from the metropolitan region of Fortaleza, state of Ceará, Brazil.

**Methods:**

This was a descriptive, quantitative, and exploratory study conducted at a primary care unit in the metropolitan region of Fortaleza, Ceará, from January to March 2019. The study population was composed of 38 health care professionals from the primary care unit. The following questionnaires were applied to obtain the situational diagnosis: the World Health Organization Disability Assessment Schedule and the Occupational Health Questionnaire.

**Results:**

Most participants were women (89.47%) and community health agents (18,42%). There were negative impacts on health conditions, such as work-related physical and mental discomfort, which was evidenced by sleep problems, sedentary lifestyle, poor access to health care services, and type of physical activity, which difer in terms of function and hierarchical levels in the field of work.

**Conclusions:**

This study showed that the questionnaires provide useful inputs regarding occupational health through the situational diagnosis and adequately address the health-disease process, as seen in primary care workers. Comprehensive care, comprehensive worker health surveillance, and participatory administration of health services should be optimized.

## Abstract (pt)

**Introdução:**

O campo da saúde do trabalhador no âmbito do Sistema Único de Saúde suscita a necessidade de revitalizar seu espaço no que condiz a coordenação do cuidado na Atenção Primária com base nos determinantes sociais em saúde.

**Objetivos:**

Contextualizar e descrever o diagnóstico situacional em saúde dos trabalhadores da atenção primária da região metropolitana de Fortaleza, Ceará.

**Métodos:**

Pesquisa descritiva, quantitativa e exploratória realizada em uma Unidade Básica de Saúde na região metropolitana de Fortaleza, Ceará, entre o período de janeiro a março de 2019. A população constituiu-se de 38 profissionais de saúde da referida unidade, através da aplicação de dois questionários para subsidiar o diagnóstico situacional: o World Health Organization Disability Assessment Schedule e o Questionário de Saúde Ocupacional.

**Resultados:**

Mediante a contextualização dos trabalhadores, foi identificado um maior número de mulheres trabalhadoras (89,47%), destacando-se, principalmente, as agentes comunitárias de saúde (18,42%), evidenciando-se impactos nas condições de saúde, como desconfortos físicos e mentais associados ao trabalho, expressos em problemas de sono, sedentarismo, pouco acesso aos serviços de autocuidado em saúde e modalidades de atividade física, que divergem quanto a função e níveis hierárquicos no campo do trabalho.

**Conclusões:**

Esta pesquisa demonstrou que os inquéritos utilizados resultam em subsídios à saúde ocupacional, por intermédio do diagnóstico situacional, além de dialogar com a problemática do processo saúde/doença, como percebidos nos trabalhadores da atenção primária. Observa-se a necessidade de otimizar a integralidade da assistência, a vigilância em saúde do trabalhador de caráter ampliado e a gestão participativa dos serviços de saúde.

## INTRODUCTION

Worker’s health is a major subject within collective health – which is also called occupational health or health and work – aimed at investigating the relationship between work activities and the health-disease process. Work is considered a fundamental part of life and social organization that determines living/health conditions.^1^

Regarding workers’ health, debates about the organization of primary care in the Brazilian Unified Health System (Sistema Único de Saúde, SUS) are related to “actions of prevention and assistance, including surveillance and intervention in the workplace, with input from workers.”^2^ Situational diagnosis, as a study instrument, seeks to support health surveillance practices that promote workers’ health, shifing the current focus on informal notifications, which does not evaluate the impact of work on workers’ health.^3^

This study innovates by assessing the health conditions of primary care workers (PCWs) from a municipality in the metropolitan region of Fortaleza, state of Ceará, Brazil, with the goal of promoting awareness among health administrators and the perception of care and self-care among PCWs, as well as to obtain a situational diagnosis of the study population. This study is also of great interest to the scientific community and the municipal government The objectives of this study were to contextualize the health conditions of PCWs and describe the health-related situational diagnosis of this population in the metropolitan region of Fortaleza, Ceará.

## METHODS

This was a descriptive, quantitative, and exploratory study conducted in the municipality of São Gonçalo do Amarante, Ceará, located in the metropolitan region of Fortaleza (state capital). Upon agreement of the unit administrator and considering a dense coverage area, the Maria Moreira de Azevedo primary care unit (PCU) (Center I) was chosen as the study site. Pre-tests were conducted in January 2019, and data collection occurred in February and March 2019 (3 months total). The study was approved by the Research Ethics Commitee of Escola de Saúde Pública do Ceará, Brazil (approval no. 3.097.134).

There was a total of 43 workers, of which 38 were included in the study. Tree workers refused to participate and two were excluded afer the eligibility criteria were applied. We included participants who worked at the Maria Moreira de Azevedo PCU (Center I). Tose who were < 6 months on the current position or who were on leave for > 6 months were excluded from the study.

The World Health Organization Disability Assessment Schedule 2.0 (WHODAS 2.0) and the Occupational Health Questionnaire (QSO) were applied, and both provided inputs for the situational diagnosis. The WHODAS 2.0 is an assessment instrument developed by the World Health Organization (WHO) to provide a standardized method for measuring health in biopsychosocial aspects. We applied the self-administered version of WHODAS 2.0, with 12 items.^4^,^5^ Scores range from 1 to 5, according to the following classification: none = 1, mild = 2, moderate = 3, severe = 4, extreme = 5. The scores are added without recoding or collapsing of response categories.^5^ The WHODAS 2.0 includes a comprehensive set of International Classification of Functioning, Disability and Health (ICF) items that are suficiently reliable and sensitive to measure the diference made by a given intervention, validated by a cross-sectional translation.^6^,^7^

The QSO was based on ergonomic criteria defined by Couto and Cardoso’s Ergonomic Census,^8^ including variables related to physical activity, occupation, level of education, working hours, sleep characteristics, and access to health care services. Descriptive analyses were conducted with relative and gross frequencies, means and standard deviations. Quantitative data were recorded in a Microsof Ofice 2013 spreadsheet.

## RESULTS

### CHARACTERIZATION OF PCU WORKERS

Table 1 reveals the predominance of young adults, aged between 26 and 35 years old (39.5%), and women (89.47%) in the study population.

**Table 1:** Sample description according to sex, age, and occupation, 2019

Data	n	%
Age (years)		
26 to 35	15	39.47
36 to 45	8	21.06
46 to 55	13	34.21
56 to 65	2	5.26
Sex		
Female	34	89.47
Male	4	10.53
Occupation		
Community health agent	7	18.42
Nursing technician/assistant	5	13.16
Administration-related positions	6	15.79
Nurse	3	7.89
Dentist	2	5.26
Family Health Strategy-related positions (physical educator, occupational therapist, nutritionist, psychologist, and social worker)	5	13.16
Cleaning/cleansing and sterilization technician	3	7.89
Dental technician/assistant	3	7.89
General service assistant	2	5.26
Endemic disease control agent	2	5.26
Time of employment (years)		
6 to 5 months	14	36.84
6 to 10	10	26.32
11 to 15	4	10.53
16 to 20	4	10.53
> 21	6	15.78
Working hours		
8	32	84.21
4	6	15.79

Most participants were Community Health Agents (CHAs) (18.42%), all of them female, followed by ofice workers (15.79%), which included four receptionists, one typewriter, and one administrative agent. Nursing technicians/assistants accounted for 13.16% of participants.

Additional information on time on the job at the PCU varied greatly: participants had been from 6 months to 5 years on the job (36.84%), with a workload of 8 hours per day.

Table 2 shows that workers who live in the metropolitan region (60.52%) spend an average of 5 to 30 minutes commuting (home-work), whereas those who live in another municipality usually spend > 30 minutes or > 1 hour commuting.

**Table 2:** Commuting characteristics (home/work, type of commuting, rest, 2019)

Data	n	%
Commuting time		
5 to 30 minutes	23	60.52
31 minutes to 1 hour	11	28.94
> 1 hour	2	5.27
Not informed	2	5.27
Type of commuting		
Walking	13	34.21
Car	12	31.57
Motorcycle	7	18.42
Bicycle	4	10.53
Bus or minibus	2	5.27
Resting area		
Yes	2	5.27
No	36	94.73
Other resting area		
Yes	11	28.95
No	24	63.15
Did not answer	3	7.90
Resting place		
Home	16	42.10
Workplace	4	10.53
Other (outside the workplace)	9	23.69
Did not answer	5	13.15
No	4	10.53

Walking was the most common type of commuting (34.21%), followed by the employers’ own car or a car rotation system (31.57%), reflecting an urban mobility characteristic of the metropolitan region of Fortaleza, Ceará. Additionally, most employees (94.73%) reported a lack of resting areas for breaks in the workplace, and 63.15% said they did not take breaks in another area (outside the workplace).

### OCCUPATIONAL HEALTH AMONG PCU WORKERS

Table 3 shows that 47.36% of respondents reported feelings of discomfort related to the activities they perform at the PCU and 50% reported sleep problems, which persisted weekly in 21.06% of them. However, because 50% of participants did not answer this item, there was not enough information to support a broader discussion about sleep frequency. Although the right amount of sleep varies according to each person, our results highlight aspects of sleep health in relation to the amount of restorative sleep.

**Table 3:** Aspects of workers’ health, 2019

Data	n	%
Body discomfort		
Neck	15	39.47
Shoulder	16	42.10
Arms	5	13.15
Elbows	1	2.63
Forearms	2	5.27
Wrist	4	10.52
Hands	3	7.89
Spine	9	23.69
Hip	8	21.05
Thighs	1	2.63
Knees	8	21.05
Legs	8	21.05
Ankles	9	23.69
Other	2	5.27
No discomfort	2	5.27
Discomfort related to your current job		
Yes	18	47.36
No	12	31.58
Not informed	8	21.06
Sleep problems		
Yes	19	50.00
No	17	44.73
Not informed	2	5.27
Frequency of sleep problems		
Daily	5	13.15
Weekly	8	21.06
Monthly	5	13.15
Every 6 months	0	0.00
Yearly	1	2.64
Did not answer	19	50.00
Hours of sleep (typical night of sleep)		
≥ 10	0	0.00
8 to 9:30	7	18.42
6 to 7:30	23	60.52
4 to 5:30	7	18.42
< 4	1	2.64

Table 4 describes access to health care services and health practices among PCU workers.

**Table 4:** Access to health care services among primary care unit workers, 2019

Data	n	%
Frequency of visits to health care services		
Never	1	2.64
Daily	1	2.64
Weekly	0	0.00
Monthly	13	34.21
Every 6 months	14	36.84
Yearly	9	23.68
Type of health care service		
Public	17	44.74
Private	6	15.78
Both	15	39.48
Frequency of physical activity		
Never	18	50.00
Daily	5	13.16
Weekly	7	18.40
Monthly	3	7.89
Every 6 months	1	2.60
Yearly	1	2.60
Not informed	2	5.26
Type of exercise		
Walking	9	23.69
Cycling	1	2.63
Muscle strengthening	4	10.52
Soccer	1	2.63
Pilates	1	2.63
Home exercises	1	2.63
Not informed	21	55.27

Table 4 shows that 36.84% of respondents seek health services every 6 months, mostly in the public sector (80%). Only 18.40% of participants practice physical exercise weekly (mostly walking and muscle strengthening), and 2.63% practice physical exercise at home.

### CHARACTERIZATION OF FUNCTIONALITY ACCORDING TO THE WHODAS 2.0

Table 5 shows the WHODAS 2.0 scores and the standard deviation. We evaluated 36 out of 38 participants because two participants did not answer all 12 items. According to the WHODAS 2.0 manual, the simple scoring allows only one unanswered item per participant. The manual also recommends that values be reported with 2 decimal points.

**Table 5:** Health conditions of workers according to WHODAS 2.0 scores and degree of difficulty, 2019

Domains	Total*	Mean†	Standard deviation
S1 – Standing (30 minutes)	77	2.13	0.83
S2 – Taking care of household responsibilities	79	2.19	0.95
S3 – Learning a new task	64	1.77	0.82
S4 – Participating in community activities	66	1.83	0.99
S5 – Emotionally affected by health problems	91	2.52	0.91
S6 – Concentration (10 minutes)	73	2.02	0.87
S7 – Walking long distances (1 km)	81	2.25	1.04
S8 – Washing your whole body	50	1.38	0.73
S9 – Getting dressed	50	1.38	0.73
S10 – Dealing with people you do not know	56	1.55	0.81
S11 – Maintaining a friendship	53	1.47	0.74
S12 – Day to day work	65	1.80	0.95

* Sum of the World Health Organization Disability Assessment Schedule 2.0 (WHODAS 2.0) items.

† None = 1; mild = 2; moderate = 3; severe = 4; extreme = 5.

Values were rounded according to data proximity. Eight domains had a mean of 2 points, which is characterized as mild difficulty. Followed by a satisfactory result, three domains did not present any difficulties in aspects related to body hygiene, dressing, or maintaining a friendship. Only one domain (emotionally affected by health problems) had a significant increase, with 3 points (moderate degree) of the rounded mean (S5).

## DISCUSSION

The WHODAS 2.0, a health instrument sufficiently sensitive to investigate the objectives of this study, allowed us to conduct a situational analysis of the degree of difficulty in general health-related activities among PCU workers in São Gonçalo do Amarante, as well as contextual factors. Diseases and illnesses, short and long-term health problems, specific bodily injuries (which were identified by the QSO), and mental/emotional problems were shown to be associated with work activities and the health-disease process.

Based on the characteristics of the study population, we identified a demand for the theme of “feminization of the aging population”, in contrast to the consequences of the increased presence of older women in the Brazilian labor market, especially in health-related jobs.^9^

Study participants, with their occupational differences and hierarchical models in terms of function and access to health care, follow a fragmented model based on stagnant programs that do not promote comprehensive care, that is, contrary to what is advocated by the Family Health Strategy (FHS). The magnitude of the programmatic and biomedical care model has not been substantially affected, as evidenced by the workers’ statements, which highlight the adverse sociocultural context.^10^ More importantly, the fragile organization of work processes was reported by CHAs as having a significant impact on job dissatisfaction rates, as observed in the field and in the specific literature.^11^

Considering the complex work activities performed by CHAs, other limiting factors can be cited, such as deficiencies in the training process, lack of social support from colleagues and administrators, work overload, and community dissatisfaction with the SUS.^12^ CHAs live the complex and confusing reality of front-line care, unlike other workers from the PCU, due to training, income, and more protected and punctual action.

Most CHAs were women, hence the relevance of studies discussing the “double or triple working day” that comprises domestic duties and professional work, and they are often responsible for providing for the family. Gender studies about the impact of domestic work and provider responsibilities are important to understand the relationship between work activities and the health-disease process.^13^

As for the sociopolitical dimensions of the problem, the low level of mobilization on the part of workers’ organizations means that the effectiveness of social structures is limited when it comes to prioritizing workers’ health actions in state and municipal health plans that, although flawed, were being developed, and are now hampered by the Federal Government’s setbacks in the SUS.^14^

Regarding the health conditions of health care workers, it should be noted that, in addition to the key role that work plays in life and in maintaining quality of life and health, issues of safety and salubrity in the workplace are highlighted,^15^ as well as the availability of free time for rest and leisure, particularly the regulation of working hours to improve living conditions outside the workplace, as was verified in the study PCU, at the same time that health services are provided to the community.

The debate may be further expanded by discussing work-related physical discomfort, which affects a great number of health care professionals and results from repetitive movements, poor posture, and extended working hours among health care professionals with more than one job. Health-related jobs should be in accordance with general labor laws. Regarding musculoskeletal disorders, it should be noted that ergonomic risk factors contribute to “the occurrence of repetitive strain injuries and work-related musculoskeletal disorders in these professionals. Therefore, occupational physical therapy should be conducted as a preventive measure or to treat current disorders”.^16^ Occupational physical therapy should be implemented in PCUs to mitigate potential health impacts.

Ergonomically, in addition to social aspects and the prevalence of pain and/or physical discomfort among those who work in health-related environments, adequate use of the workstation, which must be closely perceived by the employees, and seeing administrators as facilitators of the health adaptation process at the PCU could mitigate physical discomfort and more severe dysfunctions. Therefore, the risks presented in this study exposed variables that provide inputs for discussions about workers’ health regarding the “identification of occupational risks related to the activity performed,”^17^ which is a major concern in occupational health.

The current sociopolitical context in Brazil regarding universal health care should be taken into consideration, given the implications of the changes introduced by the National Policy of Primary Care, recently formulated in 2017, which “promote the relativization of universal coverage, the segmentation of access, the reformulation of health teams, the reorganization of the work process, and the weakening of the national policy coordination”.^18^

Still within this context, based on the understanding of worker assistance in the collective setting and as users, the multidisciplinary Family Health Support Center team, on this premise, expanded the concept of FHS to comprise the “conduction of educational or therapeutic groups by life cycle or health condition”,^19^ which is the most used health strategy among those seeking integrated care in this territory.

According to the WHO,^20^ functionality and disability are terms that denote aspects (positive and/or negative) from a biological, individual, and social perspective. Thus, the ICF provides a biopsychosocial approach with multiple perspectives, which is reflected on the multidimensional model, with dynamic interactions between individual health conditions, environmental factors, and personal factors, impacting the health of workers.

To promote the use of the ICF, alternative instruments for subsidization of data in epidemiological studies and reduction of collection time had to be developed. The instruments made available by the WHO include the Core Sets, the Checklist, the Model Disability Survey (MDS), and the WHODAS 2.0,^21^ with the latter making the subsequent analysis of the situational diagnosis of the target population more feasible. The WHODAS 2.0 domains can be interdependent, that is, each respondent has a representation that differs from the other respondents due to their individuality, which is represented by the standard deviation (Table 5).

One of the limitations of the 12-item version of WHODAS 2.0 is the lack of an established cutoff point, unlike other versions of the instrument. The WHODAS 2.0 manual states that simple scoring is specific to the sample at hand, with no weighting of individual items. Consequently, the results demonstrated by the mean values stand out in the general analysis of the data, identifying the degree of difficulty related to health conditions.^5^ A recent study that applied the WHODAS 2.0 on FHS workers showed that the WHODAS 2.0 metric could be applied as a screening instrument of functionality in the FHS, but requires cultural and terminological adaptation of terms and words”.^22^

The Primary Care Notebook (Caderno de Atenção Primária), which discusses workers’ health,^23^ describes situational diagnosis as a tool that helps to identify productive aspects and health conditions related to workers in a given territory, which is one of the first steps when implementing the FHS, with the goal of understanding the sociodemographic, environmental, epidemiological, and care profiles, as well as the needs and the health-disease process of the population.

To support the conduction of situational diagnosis in the context of workers’ health, data from the WHODAS 2.0 questionnaire, combined with the QSO, were essential for the purpose of this study. We were able to obtain a general biopsychosocial perspective, which aimed to clarify occupational and individual aspects related to primary care jobs, among other issues, especially regarding the functionality of health care professionals included in the evaluated domains of WHODAS 2.0.

## CONCLUSIONS

The health surveys used in this study provided inputs for our proposed objectives. The surveys also allowed us to characterize the profile of health care professionals at the chosen PCU and further expand the discussion of the health-disease process observed in occupational health at the municipal level of the health care network. The situational diagnosis exposed the need for awareness, comprehensive care, and participatory administration, which would benefit workers and the population.

In summary, the results showed that working women, especially CHAs, as well as other occupations, such as the reference team and the administrative sector, have high levels of sedentary lifestyles, making them more prone and vulnerable to physical discomfort. In addition, the WHODAS 2.0 revealed difficulties related to mental health conditions due to the sensitivity of the biopsychosocial aspects.

Therefore, our contribution to health studies of health care professionals is the discussion on how to prevent future work-related health problems, which was possible by outlining the profile of workers’ health problems, especially the specific care that should be provided to CHAs. Finally, we exposed important variables to support elements of occupational health surveillance, which could be used as a model for other PCUs.
